# Sleep quality mediates the relationship between problematic social media use and attention-deficit/hyperactivity symptoms

**DOI:** 10.1192/j.eurpsy.2024.250

**Published:** 2024-08-27

**Authors:** L. R. Paulina, I. Csejtei, M. Miklósi

**Affiliations:** ^1^Department of Psychiatry and Psychotherapy; ^2^Department of Clinical Psychology, Semmelweis University; ^3^Department of Media and Communication, Eötvös Loránd University; ^4^Fay Andras Foundation, OTP; ^5^Institute of Psychology, Eötvös Loránd University; ^6^Mental Health Center for Children and Adolescents, Heim Pál National Pediatric Institute, Budapest, Hungary

## Abstract

**Introduction:**

Commencing in 2019, the onset of the COVID-19 pandemic prompted an upsurge in online engagement, drawing attention to the advantages and perils associated with the use of social media. Existing research emphasizes that elevated symptom levels of attention deficit/hyperactivity disorder (ADHD) are linked not to the extent (time) of usage but to its addictive nature. However, scant research has explored its relationship with sleep quality.

**Objectives:**

In this study, we scrutinized the correlation between problematic social media usage, sleep quality, and ADHD symptoms in a non-clinical sample of young individuals during the third wave of the pandemic.

**Methods:**

We administered an online survey to 139 participants (mean age: 21.37 years, standard deviation: 2.68 years, range: 15-27). The survey encompassed various assessments, including the Bergen Social Media Addiction Scale (BSMAS), the Athens Insomnia Scale (AIS), and the self-report version of the SWAN scale (Strengths and Weaknesses of ADHD Symptoms and Normal Behavior). Participants also reported on the extent of their social media use.

**Results:**

Significant distinctions emerged in the extent of social media usage between online (M=3.12; SD=1.08) and in-person educational settings (M=2.47; SD=0.78) (t(73)=6.01; p<0.001; d=0.70). While ADHD symptom levels exhibited no correlation with the extent of social media engagement, they did exhibit a significant positive correlation with problematic usage (r=0.32; p<0.001). Likewise, the extent of social media usage displayed no correlation with sleep quality; however, problematic usage was linked to poorer sleep quality (r=0.27; p=0.002). In our mediation analysis, problematic usage correlated both directly (c’=-0.61; p=0.02) and indirectly (ab=-0.36; 95% CI: -0.60 - -0.10) with heightened ADHD symptoms through diminished sleep quality (F(1,120)=21.94; p<0.001; R2=0.27).

**Conclusions:**

Our findings affirm that it is not the extent but rather the problematic nature of social media usage that assumes significance. Moreover, our results propose that problematic usage may exacerbate ADHD symptoms, not only directly but also by influencing sleep quality.

**Disclosure of Interest:**

None Declared

